# Independent predictors of depressive symptoms and social isolation on 2-year all-cause mortality among the Korean elderly in a population-based cohort study: gender differences

**DOI:** 10.4178/epih.e2022012

**Published:** 2022-01-08

**Authors:** Hyunsuk Jeong, Hyeon Woo Yim, Beom-Woo Nam

**Affiliations:** 1 Department of Preventive Medicine, College of Medicine, The Catholic University of Korea, Seoul, Korea; 2 Department of Psychiatry, Konkuk University School of Medicine, Chungju Hospital, Chungju, Korea

**Keywords:** Depression, Social isolation, Mortality, Cohort studies, Elderly

## Abstract

**OBJECTIVES:**

This study examined whether depressive symptoms and social isolation were independent predictors of 2-year all-cause mortality among the elderly using data from a population-based cohort study.

**METHODS:**

In total, 1,033 participants (320 men and 713 women) older than 60 years of age participated in this study. Depressive symptoms, social isolation status, and socio-demographic and health-related covariates were assessed at baseline. The primary outcome measure was 2-year all-cause mortality. Data were collected through in-person interviews by trained interviewers. The GENMOD procedure was used to calculate relative risks (RRs).

**RESULTS:**

Of the 1,033 participants, 102 (40 men and 62 women) died within the follow-up period of 2 years. During the 2-year follow-up period, 17.8% of depressed men and 12.3% of depressed women died, and 29.8% of socially isolated men and 14.9% of socially isolated women died. Social isolation was an independent predictor of mortality in elderly men (adjusted relative risk [aRR], 4.6, 95% confidence interval [CI], 2.0 to 10.2), while depressive symptoms were an independent predictor of mortality in elderly women (aRR, 2.0; 95% CI, 1.2 to 3.6) when controlling for potential confounding factors. However, the depressive symptoms detected using the geriatric depression scale were not associated with mortality in men, and social isolation was not associated with mortality in women.

**CONCLUSIONS:**

The effects of depressive symptoms and social isolation on 2-year all-cause mortality within an elderly population differed according to gender. Gender-specific community-based interventions must be developed to potentially reduce 2-year all-cause mortality among the elderly.

## GRAPHICAL ABSTRACT

[Fig f1-epih-44-e2022012]

## INTRODUCTION

The proportion of individuals aged 65 years and older is expected to increase from 14.9% in 2019 to 40.1% in 2060 in Korea [1]. This rapid increase in the elderly population will have a significant influence on Korean society and will require the elderly population to be prioritized.

Depression is common and remains a significant problem for the elderly, with an estimated worldwide prevalence of 4% to 9% [2]. In Korea, the prevalence of depression is 19.9% among adults aged 65 years and older [3]. Being diagnosed with a serious medical illness or suffering from a disability increases a person’s vulnerability to depression [4]. Psychosocial adversity (e.g., economic impoverishment, social isolation, relocation, caregiving duties, and bereavement) contributes to an increased susceptibility to depression or can trigger depression in the already-vulnerable elderly population [5]. Depression in the elderly is associated with emotional suffering, increases in health expenditures, morbidity, a high risk of suicide, and increased mortality from other causes [6].

The number of community-dwelling the elderly who live alone has increased globally. According to the Ministry of Health and Welfare, 19.4% of Korean elderly live alone [7]. Living alone directly impacts social isolation in the elderly. Social isolation is particularly problematic during old age due to decreasing economic and social resources, functional limitations, and changes in family structures [8]. Social isolation negatively influences physical and mental health, as well as longevity. A previous meta-analysis found that social isolation is associated with a 50% increased risk of developing dementia [9], a 30% increased risk of incident coronary artery disease or stroke [10], and a 26% increased risk of all-cause mortality [11].

Previous studies have reported that for men, living alone is associated with a poor diet, a poor self-rated health status, and a low level of assistance from their children [12], while for women, living alone is associated with financial strain, physical limitations, and a high level of assistance from their children [13]. Although women tend to be socialized to develop a larger and more active social network, potentially protecting them from social isolation and loneliness [14], women also tend to live longer than men and are therefore more likely to be affected by widowhood or assume the role of a caregiver for their spouses. Women’s friendships tend to center on intimacy and disclosure, while friendships between men tend to center on sociability and an orientation toward tasks or activities [15]. These differences raise the possibility that the pathways by which social isolation or emotional problems impact mortality risk may differ for men and women. In order to have a better understanding of depression and social isolation’s associations with mortality in the elderly, an analysis of gender differences should be undertaken for several reasons. First, life expectancy is different for men and women. Second, the prevalence of depression and social isolation differs between men and women. Third, women and men build social networks in different ways, and men’s social relationships tend to be less intimate than women’s social relationships [16]. Fourth, it is less acceptable culturally for men to express their emotions than it is for women [17].

With regard to the association between loneliness and health, men generally have more negative attitudes towards seeking care [18]. A previous study showed that lonely men are more likely to experience low life satisfaction and are less resilient than lonely women [19]. In addition, the impact of social isolation on mortality might be greater for men since they tend to have a stronger inflammatory response when they are alone than women [20]. Moreover, unhealthy lifestyle habits, including tobacco and alcohol use, are associated with loneliness [21], which could explain the stronger association between loneliness and mortality in men than in women.

High levels of depressive symptoms are an independent risk factor for mortality in community-residing elderly [22]. The mechanisms by which depressive symptoms are associated with mortality may involve restricted daily activities due to depressive symptoms, which can contribute to mortality [23]. Depressive symptoms are classified into cognitive-affective symptoms (anhedonia, negative thoughts, and hopelessness) and somatic symptoms (fatigue and insomnia). According to a previous meta-analysis, the somatic symptoms of depression are more predictive of mortality than the cognitive-affective symptoms [24]. A previous study identified gender differences in the symptomatology of depression among the general population in Korea [25]. Women had a higher prevalence of somatic symptoms, including fatigue, hypersomnia, and noticeable psychomotor retardation, than men.

Few longitudinal studies, however, have investigated whether and how depression and social isolation are associated with mortality in the elderly and whether those relationships differ by gender. Therefore, we investigated whether depressive symptoms and social isolation are independent predictors of 2-year all-cause mortality among the elderly using population-based cohort data. Since there are differences in the characteristics of individuals according to gender, the size of the effects of depressive symptoms and social isolation on mortality also vary according to gender.

## MATERIALS AND METHODS

### Participants

The sample population included all National Basic Living Security Program recipients over 60 years of age in Chungju, which is a small town located in the central region of Korea. At the time of enrollment, the entire population of the target area was 208,202. Among this population, the number of men and women aged 60 or older was 40,196 (19.3%). In 2011, 1,535 people over 60 years of age in the target area were classified as National Basic Living Security Program recipients. As a result, we excluded the elderly who were admitted or hospitalized in facilities or hospitals and were unable to communicate due to cognitive impairment. Thus, of the 1,535 National Basic Living Security Program recipients, a total of 1,262 were ultimately enrolled in the National Basic Living Security Program Beneficiary cohort in this study. Two-year follow-up surveys were conducted between July and October 2013. Of the initial 1,262,229 were lost to follow-up, comprising a net total of 1,033 participants (320 men and 713 women) who were eligible for analysis in the present study. There were no statistical differences between participants and non-participants in age or gender.

### Measurements

Data on socio-demographic characteristics and health-related factors were collected through in-person interviews conducted by trained interviewers. To ensure the reliability of the data, the interviewers were trained in interviewing techniques, evaluation, practical application of assessment tools, and research ethics at a 2-day (16-hour) workshop before conducting the interviews.

#### Socio-demographic characteristics

Socio-demographic characteristics, including age (60-74 and 75 years or older), gender, education level, and cohabitation status (lived alone or lived with others) were recorded based on the participants’ interview responses. Medical comorbidities were assessed using self-reported answers to questions about the participants’ current medications due to health conditions including hypertension and diabetes mellitus.

#### Geriatric Depression Scale-15

The 15-item Geriatric Depression Scale (GDS-15) is a self-rated scale that was developed to detect depression and assess the severity of depressive symptoms in the elderly [26]. Total possible scores for the GDS-15 range from 0 to 15, and each item is scored based on yes-or-no responses. A high score indicates more severe symptoms of depression. The GDS-15 was found to have 92% sensitivity and 89% specificity when evaluated against diagnostic criteria. The validity and reliability of the tool have been supported through both clinical practice and research [26].

The use of the Korean version of the GDS-15 confirmed that the internal consistency was excellent in terms of content and discriminant validity for screening depression among the elderly in the context of the current study. A cut-off score of 10 was used to determine the presence of depression [27]. In the current study, the participants’ depressive symptoms were self-reported with the aid of a visiting nurse who helped patients read the questionnaire from the Korean version of the GDS-15. The Cronbach’s alpha value was found to be 0.88 in this study. We used a score of 10 or higher as the threshold in our dichotomous analysis.

#### Social isolation

Social isolation was evaluated using an abbreviated version of the Lubben Social Network Scale (LSNS-6), which evaluates the social network size of community-dwelling individuals [28]. This scale consists of 6 questions, with 3 assessing family ties and 3 assessing friendships. One sample question includes, “How many relatives do you see or hear from at least once a month?” Responses are measured on a scale of 0 to 5, with 0 indicating “none,” 1 indicating “1,” 2 indicating “2,” 3 indicating “3 or 4,” 4 indicating “5 to 8,” and 5 indicating “9 or more.” The total score represents the respondent’s social network size with an equally-weighted sum of the 6 items, and the total possible scores range from 0 to 30 [28].

Higher scores represent larger family-based or friendship-based social networks. In the present study, the LSNS-6 showed acceptable internal consistency with a Cronbach’s alpha of 0.76. We used the Korean version of the LSNS-6 [29]. Social isolation can be defined structurally as the absence of social interactions, contacts, and relationships with family, friends, and neighbors on an individual level [30]. Therefore, social isolation was determined when respondents answered “none” to all 6 questions.

#### Two-year all-cause mortality

The outcome of this research was 2-year all-cause mortality. Death was determined through door-to-door visits to the participants’ homes. At the 2-year follow-up assessment, if death could not be confirmed because a participant no longer resided in the same place as at the time of registration, the participant was considered lost to follow-up.

### Statistical analysis

For the baseline characteristics of the study population, the numerical variables were expressed using mean and standard deviation, and the categorical variables were expressed using the number and proportion (%). Either the chi-square test or t-test was used to evaluate differences between men and women. We calculated all-cause mortality during the 2-year follow-up period separately for men and women. We investigated the association between depressive symptoms and all-cause mortality using the GENMOD procedure to calculate the relative risks (RRs) of 2-year all-cause mortality with a reference category of non-depressive symptoms in men and women. In the multivariable model, we adjusted for age in model 2. Model 3 included the variables from model 2 as well as cohabitation status, education level, and diagnoses of hypertension and diabetes mellitus at baseline. Similar models were created for social isolation, using a non-socially isolated group as the reference category.

Since depression and social isolation are closely related, in model 4, we showed the exploratory results of further adjustment for social networks and depressive symptoms, including the variables from model 3. The new concept of an E-value for both the observed association estimate of adjusted relative risk (aRR) and the limit of the confidence interval (CI) closest to null was calculated. The E-value is defined as the minimum strength of association that 1 or more unmeasured confounders would need to have on both the treatment and the outcome to fully explain a specific treatmentoutcome association depending on the measured covariates [31]. All analyses were conducted using SAS version 9.4 (SAS Institute Inc., Cary, NC, USA). All statistical tests were two-tailed, and the alpha value was set at 5%. In addition, the 95% CIs of the risk estimates were reported.

### Ethics statement

Informed consent was acquired from all of the participants following an explanation of the research principles, including confidentiality and the freedom of choice to participate. This study received full review and approval from the Institutional Review Board of the Catholic University of Korea (CUMC11U035). All procedures were conducted in accordance with the Helsinki Declaration. Written consent was acquired from all participants following an explanation of the research principles, including confidentiality and the freedom of choice to participate.

## RESULTS

The baseline socio-demographic characteristics of the 1,033 participants are shown in Table 1. Men and women differed significantly for most variables. There were more elderly women (69.0%) than elderly men (31.0%), and more women (58.2%) lived alone than men (45.3%). A higher proportion of women (61.4%) had hypertension than men (47.5%), and women had a higher mean score for social network size than men. Men participants had an overall higher education level than women participants (Table 1).

Of the 1,033 participants, 102 (40 men and 62 women) died within the 2-year follow-up period. In total, 11.1% of elderly men without depression at the baseline died compared to 17.8% of elderly men with depression at the baseline. In addition, 9.5% of non-socially isolated elderly men and 29.8% of socially isolated elderly men died during the 2-year follow-up period. A total of 6.3% of women without depression died within the 2-year follow-up period compared to 12.3% of women with depression. In addition, 8.3% of non-socially isolated elderly women and 14.9% of socially isolated elderly women died during the 2-year follow-up period. Excess mortality attributable to depression among depressed men was 36.4 deaths per 1,000 population and 60.3 deaths per 1,000 population among depressed women. Excess mortality attributable to social isolation among socially isolated men was 202.6 deaths per 1,000 population and 66.4 deaths per 1,000 population among socially isolated women (Table 2).

Depressed women had a 2-fold higher risk of death (aRR, 2.0; 95% CI, 1.2 to 3.6) than women who did not have significant depressive symptoms after adjusting for potential confounding variables. The point estimate E-value was 3.4 and the CI E-value was 1.7. However, depressive symptoms were not significantly associated with 2-year mortality for the men participants in this study. Socially isolated men had a 4.6-fold higher risk of death (aRR, 4.6; 95% CI, 2.0 to 10.2) than men with active social networks after adjusting for potential confounding variables. The results of additional adjustment for either social network or depressive symptoms in model 4 were not different from the results of model 3. The point estimate E-value was 8.5 and the CI E-value was 3.2. However, social isolation was not significantly associated with 2-year mortality among women (Table 3).

## DISCUSSION

In the current study, the effects of depressive symptoms and social isolation on 2-year all-cause mortality among the elderly differed according to gender. For elderly men, social isolation increased the risk of 2-year all-cause mortality by 4.6 times compared to those who were not socially isolated. However, the depressive symptoms identified by the GDS-15 were not associated with 2-year all-cause mortality in men.

Social isolation is known to affect health-related physical activity and lifestyle [32]. Socially isolated individuals engage in less physical activity and are often sedentary due to limited access to transportation, reduced contact with friends and family, and living alone [33].

Traditionally, the predominant social role of women has been that of a housewife, while the traditional social role of men is to work to provide income and material resources to support their families. Given that participants in the current study were the elderly aged 60 years or older, older men in our analysis may not have been accustomed to preparing food or doing housework on their own. Thus, elderly men who are socially isolated may be at a greater risk of malnutrition and dietary inadequacy than elderly women who are socially isolated [34].

Additionally, non-specific and broad social connectedness, as well as social isolation, have been shown to have a greater impact on the perceived health status of men than women [35]. Traditionally, men tend to pursue social connections that provide instrumental support, whereas women tend to seek emotional support from their relationships [36]. These findings are consistent with the evidence in this study. For men, depression (as determined by the GDS-15) was not associated with 2-year all-cause mortality, while social isolation was found to be highly associated with mortality. In order to improve the well-being of elderly men, social efforts must be undertaken to mitigate social isolation.

The prevalence of social isolation among elderly men in the current study was 14.7%, which has significant public health implications. According to the Comprehensive Support for the Elderly Living Alone, the elderly living alone in Korea accounted for 19.4% of the total elderly population in 2018 [7]. If social isolation can be interpreted as a substantial cause of mortality among elderly men and approximately 10% of elderly men experience social isolation, then mortality could be prevented for approximately 438 out of 10,000 (minimum 190 to maximum 971) elderly men within 2 years of the baseline by improving their social networks.

For elderly women, depression (as determined by the GDS-15) increased the risk of 2-year all-cause mortality two-fold compared to individuals without depressive symptoms. However, social isolation was not associated with 2-year all-cause mortality for women.

The prevalence of social isolation among elderly women was 6.6%, which was lower than it was among elderly men (14.7%). Elderly women who live alone tend to maintain emotional intimacy with friends and neighbors, thus maintaining social bonds better than older men [37]. For women, the effects of social isolation on physical health are less severe than they are for men since women tend to have better self-care abilities due to lifestyle habits related to traditional social roles.

As people grow old, many individuals experience social isolation due to living alone, a lack of close family ties, or an inability to participate in community activities. This phenomenon is commonly accompanied by depression. Social isolation is a common comorbidity with depression [38]. In our study, 122 elderly men had depressive symptoms, of which 23 (18.9%) were socially isolated, and a total of 284 elderly women had depressive symptoms, of which 31 (11.9%) were socially isolated.

At the time of the 2-year follow-up, 102 deaths were recorded between 2012 and 2013. To compare the mortality rate in our study with that of the general population, the standardized mortality rate (SMR) was calculated by comparing the 2012-2013 age-specific mortality rate in Korea, and the 95% CI of the SMR was obtained using the Poisson distribution method [39]. Compared to the general population, our sample had a slightly higher SMR, at 1.06 (95% CI, 0.85 to 1.31) (Supplementary Material 1). However, the difference was not statistically significant.

There is a large body of literature suggesting that women are more susceptible to depressive symptoms than men [40]. The reasons for these differences between men and women are not yet clear, but the biological and psychological differences and copying styles of men and women might be a possible explanation [41].

Physician-diagnosed depression is associated with a substantial increase in the risk of mortality, particularly death from cardiovascular disease, in women [42]. The depressive symptoms detected by the GDS-15 are broader than the diagnostic criteria for major depressive disorder. The prevalence of depressive symptoms detected by the GDS-15 in elderly women in this study was 39.8%. However, depressive symptoms in elderly women showed a two-fold association with 2-year all-cause mortality in this study.

According to a report based on the 2020 National Survey of Older Koreans, the prevalence of depressive symptoms detected by the GDS-15 within the elderly population of Korea was 13.5% [43]. If depressive symptoms can be interpreted as a cause of mortality among elderly women and if 10% of women have depressive symptoms, then mortality can be prevented for 121 out of 10,000 (minimum 72 to maximum 218) elderly women within 2 years of the baseline if their depressive symptoms are managed.

Our study has several strengths and several potential limitations that need to be acknowledged. Data on socio-demographic and health-related factors were collected through in-person interviews using an easily administered, validated, and standardized tool to detect the presence of depressive symptoms and social isolation in the elderly. Due to the observational nature of the cohort design, our analysis is vulnerable to residual confounding factors when attempting to establish causality. We calculated E-values to assess the robustness of associations with potential unmeasured or uncontrolled confounding factors. The estimated E-value for the association between social isolation and all-cause mortality in elderly men was 8.5. This suggests that, in order to determine that there is no causal relationship between social isolation and all-cause mortality in elderly men, there would need to be 1 or more unmeasured confounders that increase the risk of all-cause mortality by 8.5 times.

Moreover, the estimated E-value for the association between depressive symptoms and all-cause mortality in elderly women was 3.4. This suggests that, in order to determine that there is no causal relationship between depressive symptoms and all-cause mortality among elderly women, there would need to be 1 or more unmeasured confounders that increase the risk of all-cause mortality by 3.4 times.

These results should be considered in the context of several potential limitations. We obtained our data on elderly individuals exclusively using a sample of National Basic Living Security Program recipients to determine groups of individuals at high risk for depressive symptoms and social isolation. This should be considered when generalizing our results to other populations. Nevertheless, because this is a cohort study in which the magnitude of the effects is measured in terms of RRs, it can be safely assumed that the results will not be different for other populations.

Some symptoms of social isolation and depression overlap, which may result in an overestimation of the association between social network size and depression. However, both conditions were measured extensively using well-established and reliable instruments that do not measure overlapping symptoms. The proportion of participants who were lost to follow-up was 19% in this study. Non-participation in surveys throughout the follow-up period could be a source of bias. Regardless, this type of bias is unavoidable in longitudinal studies on aging populations that include community-dwelling elderly only. There were no differences in the baseline scores for depressive symptoms and social network size between the participants who were lost to follow-up and those who completed follow-up for both men and women. If objective data were collected to ascertain mortality, then the data could have been linked to cause-of-death data from the National Statistical Office. Although the deaths of participants were confirmed using the public health center’s list of basic livelihood benefits recipients, the accuracy of this list has not been verified, which is a limitation of this study.

A significant difference in social isolation according to gender was observed (p< 0.05). However, the differences in depressive symptoms according to gender were not statistically significant (p= 0.103). The failure of the interaction term to achieve statistical significance may be due to an inadequate sample size. Since an association between depression and gender was expected, the analysis was stratified by gender even though the findings were not statistically significant. The association between depressive symptoms and 2-year all-cause mortality in women shown in this study suggests the possibility that depressive symptoms may increase the risk of death for women more than for men. A well-designed future study should confirm an independent research hypothesis on this topic.

From a public health perspective, the findings from the current study suggest that gender-specific community-based interventions are needed to mitigate all-cause mortality among the elderly. Loneliness-reduction programs can be launched to expand elderly men’s social networks or help them develop new social networks, and social support focusing on interventions to improve the emotional security and intimacy of elderly women’s relationships may be implemented to reduce the risk of mortality in both genders. In the meantime, community healthcare professionals are advised to assess social isolation and depression to identify vulnerable the elderly who may benefit from existing interventions. In addition, the elderly with depression should be referred to psychiatric, psychological, and social interventions for appropriate treatment and to potentially reduce mortality.

## Figures and Tables

**Figure f1-epih-44-e2022012:**
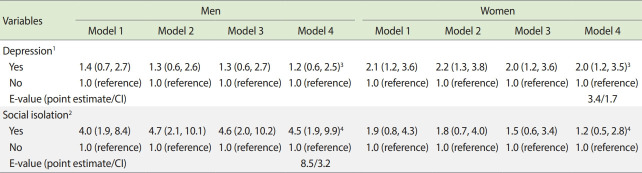


**Table 1.** Baseline socio-demographic characteristics and health status of participants (n=1,033)

Characteristics	Men	Women	p-value
Total	320 (31.0)	713 (69.0)	
Age (yr)			<0.001
60-74	220 (68.8)	380 (53.8)	
≥75	100 (31.2)	333 (46.7)	
Education level (yr)			<0.001
None	51 (15.9)	328 (46.0)	
1-6	143 (44.7)	313 (43.9)	
≥7	126 (39.4)	72 (10.1)	
Cohabitation status			<0.001
Lived alone	145 (45.3)	415 (58.2)	
Lived with others	175 (55.7)	298 (41.8)	
Hypertension	152 (47.5)	438 (61.4)	<0.001
Diabetes mellitus	74 (23.1)	160 (22.4)	0.808
Depression (GDS-15≥10)	122 (38.1)	284 (39.8)	0.604
Social isolation^1^	47 (14.7)	47 (6.6)	0.001

Values are presented as number (%). GDS-15, 15-item Geriatric Depression Scale.

1 Social isolation was defined as answers of “none” to all 6 questions.

**Table 2.** Two-year mortality rates according to baseline depression and social isolation in men and women

Predictors	Categories	Men (n=320)				Women (n=713)			
		n	Death, n (%)	Incidence per 1,000 population	Attributable risk	n	Death, n (%)	Incidence per 1,000 population	Attributable risk
Depression^1^	Yes	122	18 (17.8)	147.5	36.4	284	35 (12.3)	123.2	60.3
	No	198	22 (11.1)	111.1	Reference	429	27 (6.3)	62.9	Reference
Social isolation^2^	Yes	47	14 (29.8)	297.9	202.6	47	7 (14.9)	148.9	66.4
	No	273	26 (9.5)	95.2	Reference	666	55 (8.3)	82.6	Reference

1 Depression was defined as a score of 10 points or more on the Geriatric Depression Scale.

2 Social isolation was defined as answers of “none” to all 6 questions.

**Table 3.** Relative risk of depression and social isolation for 2-year mortality in men and women

Variables		Men				Women			
		Model 1	Model 2	Model 3	Model 4	Model 1	Model 2	Model 3	Model 4
Depression^1^									
	Yes	1.4 (0.7, 2.7)	1.3 (0.6, 2.6)	1.3 (0.6, 2.7)	1.2 (0.6, 2.5)^3^	2.1 (1.2, 3.6)	2.2 (1.3, 3.8)	2.0 (1.2, 3.6)	2.0 (1.2, 3.5)^3^
	No	1.0 (reference)	1.0 (reference)	1.0 (reference)	1.0 (reference)	1.0 (reference)	1.0 (reference)	1.0 (reference)	1.0 (reference)
	E-value (point estimate/CI)								3.4/1.7
Social isolation^2^									
	Yes	4.0 (1.9, 8.4)	4.7 (2.1, 10.1)	4.6 (2.0, 10.2)	4.5 (1.9, 9.9)^4^	1.9 (0.8, 4.3)	1.8 (0.7, 4.0)	1.5 (0.6, 3.4)	1.2 (0.5, 2.8)^4^
	No	1.0 (reference)	1.0 (reference)	1.0 (reference)	1.0 (reference)	1.0 (reference)	1.0 (reference)	1.0 (reference)	1.0 (reference)
	E-value (point estimate/CI)				8.5/3.2				

Values are are presented as relative risk (95% CI). Model 1: Crude relative risk; Model 2: Adjusted for age; Model 3: Adjusted for age, cohabitation status, education level, hypertension, and diabetes mellitus at baseline; Model 4: An exploratory analysis. CI, confidence interval.

1 Depression was defined as a score of 10 points or more on the Geriatric Depression Scale.

2 Social isolation was defined as answers of “none” to all 6 questions.

3 Adjusted for age, cohabitation status, education level, hypertension, diabetes mellitus, and social network at baseline.

4 Adjusted for age, cohabitation status, education level, hypertension, diabetes mellitus, and depressive symptom scores at baseline.

## SUPPLEMENTARY MATERIALS

Supplementary Material 1. Standardized mortality rate in 2012-2013
