# Supplementary material for: Independent predictors of depressive symptoms and social isolation on 2-year all-cause mortality among the Korean elderly in a population-based cohort study: gender differences
Source: Epidemiol Health. 2022 Jan 8;44:e2022012. doi: 10.4178/epih.e2022012 (PMC9117106; doi:10.4178/epih.e2022012)
Supplement: Supplementary Material 1. — Standardized mortality rate in 2012-2013 [file epih-44-e2022012-suppl1.docx]

Supplementary Material 1. Standardized mortality rate in 2012-2013

| Age group | No. of death for 2 years (2012-2013) within the cohort | No. of population within the cohort | Age-specific mortality rate/100,000 in Korea (2012-2013) | Age-specific mortality rate in Korea (2012-2013) | No. of expected death in standard population |
| --- | --- | --- | --- | --- | --- |
| 70-74 | 27 | 322 | 3836.2 | 0.038362 | 12.352564 |
| 75-79 | 19 | 271 | 6909.9 | 0.069099 | 18.725829 |
| 80-84 | 17 | 177 | 12428 | 0.12428 | 21.99756 |
| 85-89 | 16 | 85 | 22320 | 0.2232 | 18.972 |
| 90+ | 10 | 29 | 40301.2 | 0.403012 | 11.687348 |
| Total | 89 |  |  |  | 83.735301 |
| SMR =1.063 (95% CI: 0.854-1.308, *P*=0.297) | | | | | |
